# Live Visualization of Calcified Bones in Zebrafish and Medaka Larvae and Juveniles Using Calcein and Alizarin Red S

**DOI:** 10.21769/BioProtoc.5142

**Published:** 2024-12-20

**Authors:** Rina Koita, Sae Oikawa, Taisei Tani, Masaru Matsuda, Akinori Kawamura

**Affiliations:** 1 Division of Life Science, Graduate School of Science and Engineering, Saitama University, Shimo-Okubo 255, Sakura-ku, Saitama, Japan; 2 Center for Bioscience Research and Education, Utsunomiya University, 350 Mine-machi, Utsunomiya, Japan

**Keywords:** Zebrafish, Medaka, Calcified bones, Live staining, Calcein, Alizarin red S

## Abstract

Zebrafish and medaka are valuable model vertebrates for genetic studies. The advent of CRISPR-Cas9 technology has greatly enhanced our capability to produce specific gene mutants in zebrafish and medaka. Analyzing the phenotypes of these mutants is essential for elucidating gene function, though such analyses often yield unexpected results. Consequently, providing researchers with accessible and cost-effective phenotype analysis methods is crucial. A prevalent technique for investigating calcified bone development in these species involves using transgenic fish that express fluorescent proteins labeling calcified bones; however, acquiring these fish and isolating appropriate crosses can be time-consuming. We present a comprehensive protocol for visualizing ossified bones in zebrafish and medaka larvae and juveniles using calcein and alizarin red S staining, which is both economical and efficient. This method, applicable to live specimens during the ossification of bones, avoids apparent alterations in skeletal morphology and allows for the use of different fluorescent dyes in conjunction with transgenic labeling, thus enhancing the analysis of developmental processes in calcifying bones, such as vertebrae and fin rays.

Key features

• The calcified bones of alive zebrafish and medaka larvae and juveniles can be visualized repeatedly using simple and inexpensive calcein and alizarin red S.

• No need to use transgenic fish to visualize ossified bones, allowing for rapid analysis of bone phenotypes in mutants.

• Double staining is possible in transgenic fish with reporter genes such as *GFP* and *DsRed* using alizarin red S or calcein, which exhibit different fluorescence.

• Ossification processes of bones such as vertebrae, ribs, and fin rays can be analyzed in mutants.

## Graphical overview


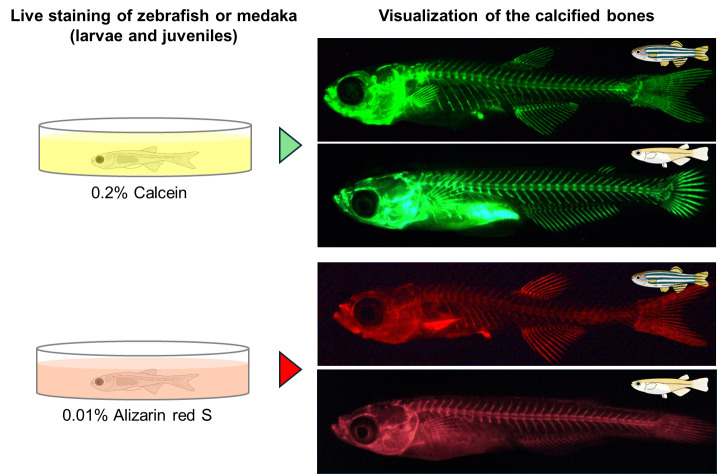


## Background

Zebrafish and medaka are model vertebrates that are amenable to genetic approaches. Chemical mutagenesis has led to the isolation of hundreds of mutants in these species [1,2], contributing greatly to our knowledge of vertebrate development and other biological phenomena. The development of CRISPR-Cas9 technology has further enhanced our ability to generate mutants of specific genes in vertebrate models [3–5]. To fully understand the function of a gene, it is crucial to analyze the phenotype of the mutants. However, the generation of mutants often results in unexpected phenotypes. Therefore, it is important to provide researchers with accessible and cost-effective methods to analyze the phenotypes.

A common approach to studying the development of calcified bones in zebrafish and medaka is to use transgenic fish that specifically express fluorescent proteins labeling calcified bones [6,7]. However, it is necessary to first obtain transgenic fish, especially for researchers who do not specialize in bone research. Even if they manage to obtain them, it takes several months to analyze them because new fish have to be isolated by crossing transgenic fish with the mutant fish. Here, we describe a detailed step-by-step protocol for visualizing the ossified bones of zebrafish and medaka larvae and juveniles. This method allows the visualization of calcified bones in zebrafish and medaka by staining with calcein and alizarin red S, which are inexpensive reagents. The staining can be performed in one or two days on live zebrafish and medaka and can be repeated multiple times, as it does not cause any noticeable abnormalities in the subsequent skeletal morphology such as vertebrae and fin rays [8,9]. In addition, calcein and alizarin red S are different fluorescent dyes, and double staining is possible by using different fluorescent dyes depending on the fluorescent protein in transgenic fish. Analysis of mutants using this method is expected to improve our understanding of various developmental processes in the calcifying bones of zebrafish and medaka, including vertebrae and fin rays.

## Materials and reagents

**Biological materials**

1. Zebrafish (*Danio rerio*, Riken Wild-type (RW) strain, the National BioResource Project Zebrafish in Japan)
2. Medaka (*Oryzias latipes*, Hd-rR strain, the National BioResource Project Medaka in Japan)

**Reagents**

1. Calcein (Fujifilm Wako, catalog number: 340-00433)
2. NaOH (Fujifilm Wako, catalog number: 194-18865)
3. NaCl (Fujifilm Wako, catalog number: 191-01665)
4. KCl (Fujifilm Wako, catalog number: 160-03555)
5. CaCl_2_ (Fujifilm Wako, catalog number: 038-19735)
6. HEPES (Fujifilm Wako, catalog number: 342-01375)
7. Alizarin red S (Fujifilm Wako, catalog number: 011-01192)
8. KOH (Fujifilm Wako, catalog number: 168-21815)
9. Tricaine (Fujifilm Wako, catalog number: 051-06571)
10. 2-Phenoxyethanol (Fujifilm Wako, catalog number: 163-12075)
11. Methylcellulose 1500 cP (Fujifilm Wako, catalog number: 139-02145)

**Solutions**

1. 2.0% calcein stock solution (see Recipes)
2. 0.2% calcein staining solution (see Recipes)
3. 1× Ringer’s solution (see Recipes)
4. 1/3× Ringer’s solution (see Recipes)
5. 0.1% alizarin red S stock solution (see Recipes)
6. 0.01% alizarin red S staining solution (see Recipes)
7. Tricaine (MS-222) anesthetizing stock solution for zebrafish (see Recipes)
8. 2.0% methylcellulose solution (see Recipes)

**Recipes**

1. **2.0% calcein stock solution**
  **Table:** 

Reagent	Final concentration	Amount
Calcein	2.0%	1 g
0.5 N NaOH solution	Adjust to pH 7.0	
1/3 Ringer’s solution (Recipe 4)	n/a	Add up to 50 mL
Total	n/a	50 mL
  The prepared 2.0% calcein stock solution should be aliquoted into small tubes and stored at -20 °C in the dark. Instead of 1/3 Ringer’s solution, E3 or similar buffers can be used. Prepare a 0.5 N NaOH solution by dissolving 1.0 g of NaOH in 50 mL of distilled water.
2. **0.2% calcein staining solution**
  **Table:** 

Reagent	Final concentration	Amount
2.0% calcein stock solution (Recipe 1)	0.2%	300 μL
1/3 Ringer’s solution (Recipe 4)	n/a	2.7 mL
Total	n/a	3.0 mL
  0.2% calcein staining solution should be prepared prior to staining. The example shown above is for a 35 mm dish; the volume to be added is 3.0 mL. If more dishes are used, the volume can be increased. Instead of 1/3 Ringer’s solution, E3 or similar buffers can be used.
3. **1× Ringer’s solution**
  **Table:** 

Reagent	Final concentration	Amount
NaCl	116 mM	6.78 g
KCl	2.9 mM	0.216 g
CaCl_2_·2H_2_O	1.8 mM	0.265 g
HEPES	5.0 mM	1.192 g
0.5 N NaOH solution	Adjust to pH 7.0	
DW	n/a	Add up to 1,000 mL
Total	n/a	1,000 mL
  1× Ringer’s solution is stable at room temperature for years.
4. **1/3× Ringer’s solution**
  **Table:** 

Reagent	Final concentration	Amount
1× Ringer’s solution (Recipe 3)	1/3×	333 mL
DW	n/a	Add up to 1,000 mL
Total	n/a	1,000 mL
  1/3× Ringer’s solution is stable at room temperature for years.
5. **0.1% alizarin red S stock solution**
  **Table:** 

Reagent	Final concentration	Amount
Alizarin red S	0.1%	0.05 g
1 N KOH solution	Adjust to pH 7.4	
1/3 Ringer’s solution (Recipe 4)	n/a	Add up to 50 mL
Total	n/a	50 mL
  0.1% alizarin red S stock solution should be stored at room temperature in the dark. Instead of 1/3 Ringer’s solution, E3 or similar buffers can be used. Prepare a 1 N KOH solution by dissolving 2.0 g of KOH in 50 mL of distilled water.
6. **0.01% alizarin red S staining solution**
  **Table:** 

Reagent	Final concentration	Amount
0.1% alizarin red S stock solution (Recipe 5)	0.01%	300 μL
1/3 Ringer’s solution (Recipe 4), or DW	n/a	2.7 mL
Total	n/a	3.0 mL
  0.01% alizarin red S staining solution should be prepared prior to staining. The example shown above is for a 35 mm dish; the volume to be added is 3.0 mL. If more dishes are used, the volume can be increased. Instead of 1/3 Ringer’s solution, E3 or similar buffers can be used.
7. **Tricaine (MS-222) anesthetizing stock solution for zebrafish**
  **Table:** 

Reagent	Final concentration	Amount
Tricaine	2.0%	1 g
DW		Add up to 50 mL
Total	n/a	50 mL
  The prepared tricaine anesthetizing stock solution should be divided into small tubes and stored at -20 °C in the dark.
8. **2.0% Methylcellulose solution**
  **Table:** 

Reagent	Final concentration	Amount
Methylcellulose 1500 cP	2.0%	1 g
DW	n/a	Add up to 50 mL
Total	n/a	50 mL
  To completely dissolve the methylcellulose in DW, shake vigorously for several days. When photographing, the fish is placed in this viscous solution to orient the fish in the desired position. It is possible to adjust the solution to a percentage of 2%–3% methylcellulose depending on personal preference.

**Laboratory supplies**

1. Non-treated dish, 35 mm (IWAKI, catalog number: 1000-035)
2. Glass-base dish, 35 mm (IWAKI, catalog number: 3970-035)
3. Transfer pipette, 3 mL (BD Falcon, catalog number: 357575)

## Equipment

1. Fluorescent stereomicroscope (Leica, model: M205 FA)
2. Digital camera (Leica, model: DFC350 FX)

## Software and datasets

1. ImageJ (v. 1.53e, September 2020, free to use, https://imagej.net/ij/)

## Procedure

Both calcein (green fluorescent signal) and alizarin red S (red fluorescent signal) can be used to visualize calcified bones in live zebrafish and medaka. However, calcein is recommended, except in cases such as staining the bones of transgenic fish with the *GFP* gene. Calcein has a shorter staining time, higher signal-to-noise ratio, and stronger fluorescent intensity than alizarin red S. Visualization of the bones in the internal body, such as vertebrae, is possible until the scales have calcified. In the following protocol, staining methods are essentially the same for zebrafish and medaka, unless otherwise noted. When referring to “fish,” both zebrafish and medaka are included in this protocol.

- **Calcein staining in live zebrafish and medaka larvae and juveniles**
  1. Obtain fertilized embryos by crossing female and male adult fish.
  2. Raise the fish embryos to the desired developmental stages for analysis. For more information on the developmental stages of zebrafish and medaka, see [10–12].
  3. Use a 35 mm dish for calcein staining. Each dish can accommodate up to five fish for staining. To ensure an even staining, avoid placing more than six fish in one dish. Prepare the necessary number of 35 mm plastic dishes.
  4. Prepare 3 mL of 0.2% Calcein staining solution per dish to be analyzed. Adjust the volume of the staining solution based on the number of dishes. After preparation, store the solution at room temperature in the dark.
  5. In addition, make sure to have plenty of room-temperature water (possibly with 1/3 Ringer’s solution or tap water that has been left for at least one day to remove sodium hypochlorite) for washing purposes.
  6. First, place the fish whose bones need to be visualized in a 35 mm dish filled with enough water. When transferring the fish, use a 3 mL transfer pipette with the tip removed, allowing the larvae to pass through without damage. Carefully transfer the fish to the 35 mm dish.
  7. Next, remove water using a regular 3 mL pipette (uncut tip). Shift the lid slightly to prevent the fish from jumping while removing the water.
  8. Immediately add 3 mL of 0.2% Calcein staining solution to the 35 mm dish and stain the fish at room temperature for 15 min. Cover the dish with a lid to prevent the fish from jumping during staining (Figure 1A).
    **Figure 1.**
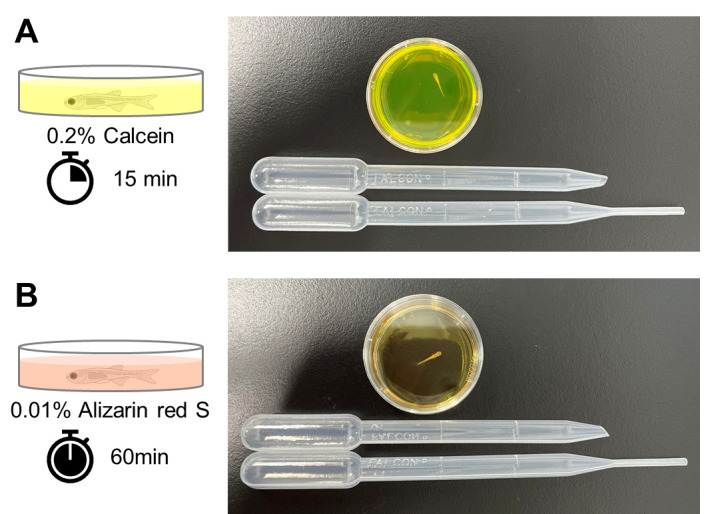
Staining of calcified bones in live juvenile zebrafish. To stain the ossified bones, a live juvenile zebrafish is placed in a 35 mm dish and stained with calcein (A) or alizarin red S (B). For transferring the fish, a 3 mL transfer pipette with the tip cut off is used, while a separate 3 mL transfer pipette with the tip intact is used to transfer the solution.
  9. Discard the 0.2% Calcein staining solution using a 3 mL transfer pipette. Immediately add approximately 4 mL of water to the 35 mm dish and mix gently.
  10. Remove the water using a 3 mL transfer pipette. Immediately add another 4 mL of water into the 35 mm dish and mix gently. Place the fish in the 35 mm dish for 5 min. Repeat this process three times until the yellow dye of the calcein disappears.
  11. Keep the stained fish in water in the 35 mm dishes. Fluorescent images can be captured immediately after staining. However, it is recommended that pictures be taken the day after staining to reduce background signals and obtain high signal-to-noise ratio images. When photographing the next day, place the fish in an environment with the optimum temperature and do not feed them until the photographs have been taken.
  12. Stained calcein signals can still be detected in areas that were calcified at the time of the initial procedure, even after 3 days. The pulse-chase experiments utilizing dual labeling with alizarin red S allow for the analysis of the ossification process in the bones of interest. For details, see Akama et al. [9].
- **Alizarin red S staining of zebrafish and medaka larvae and juveniles**
  1. The alizarin red S staining method is basically the same as calcein staining, except for the staining time.
  2. Perform the same procedures as described in steps A1–7.
  3. Immediately add 3 mL of 0.01% alizarin red S staining solution to the 35 mm dish and stain the fish at room temperature for 60 min. Cover the dish with a lid to prevent the fish from jumping during staining (Figure 1B).
  4. Perform the same procedures as described in steps A9–11.
- **Anesthesia treatment of zebrafish and medaka prior to imaging**
  1. Administering anesthesia to zebrafish and medaka larvae and juveniles is a critical and potentially risky step in this protocol, so caution must be exercised to avoid overdosing the fish. It is recommended to administer anesthesia to one fish at a time just before capturing fluorescent images. The amount of anesthesia required depends on the size of the fish, so it is advisable to anesthetize the smaller fish first.
  2. For zebrafish, prepare a new 35 mm dish by adding 3 mL of water and 10 μL of tricaine in the room with the fluorescent stereomicroscope. For medaka, prepare a new 35 mm dish by adding 3 mL of water and 3 μL of 2-phenoxyethanol.
  3. Prepare the stained fish in 35 mm dishes.
  4. For fluorescence imaging, prepare a 35 mm glass-base dish filled with a 2% methylcellulose solution in the glass section.
  5. Using a 3 mL transfer pipette with the tip removed, carefully transfer one fish to the 35 mm dish with the anesthesia.
  6. Ensure that the fish are immobilized by the anesthesia treatment. If the fish are still moving, add more anesthesia reagents until they are still. Again, it is important to be cautious with the amount of anesthesia given, as excessive amounts of anesthesia can be fatal for larvae and juveniles. The anesthetic solution can be used for other fish repeatedly.
  7. Using a 3 mL transfer pipette with the tip removed, carefully transfer one fish to a 35 mm glass base dish filled with 2% methylcellulose solution for imaging.
- **Imaging of stained ossified bones of zebrafish and medaka using a fluorescent stereomicroscope**
  1. Zebrafish and medaka grow to over 5 mm in length during the ossification of their skeletons. Fluorescent images of stained calcified bones can be observed using a fluorescent stereomicroscope. It is important to note that calcein or alizarin red S staining does not cause obvious developmental abnormalities in zebrafish and medaka, so the fish can be recovered and grown normally after the photographs are taken. Additionally, calcein signals can be captured under the same conditions as normal GFP signals, while alizarin red S signals can be captured under the same conditions as normal DsRed signals.
  2. When transferring anesthetized fish to a 2% methylcellulose solution in the 35 mm dish, it is important to be aware that the anesthesia wears off after several minutes, causing the fish to start moving. Therefore, it is crucial to efficiently capture the desired fluorescent images within a limited time. If the fish begin to move during the analysis, it is advisable to re-anesthetize them.
  3. The standard length (SL), which is the length of the fish from the tip of the snout to the posteriormost region of the body where caudal fin rays insert, is typically used to indicate the developmental stages of larvae and juvenile fish [12]. Thus, it is crucial to start by capturing a clear image of the entire stained fish. Position the anesthetized fish, include a scale bar for reference, and take a lateral image of the entire fish.
  4. Capture the desired fluorescent images by adjusting the orientation of the stained fish and changing the magnification (Figures 2, 3).
    **Figure 2.**
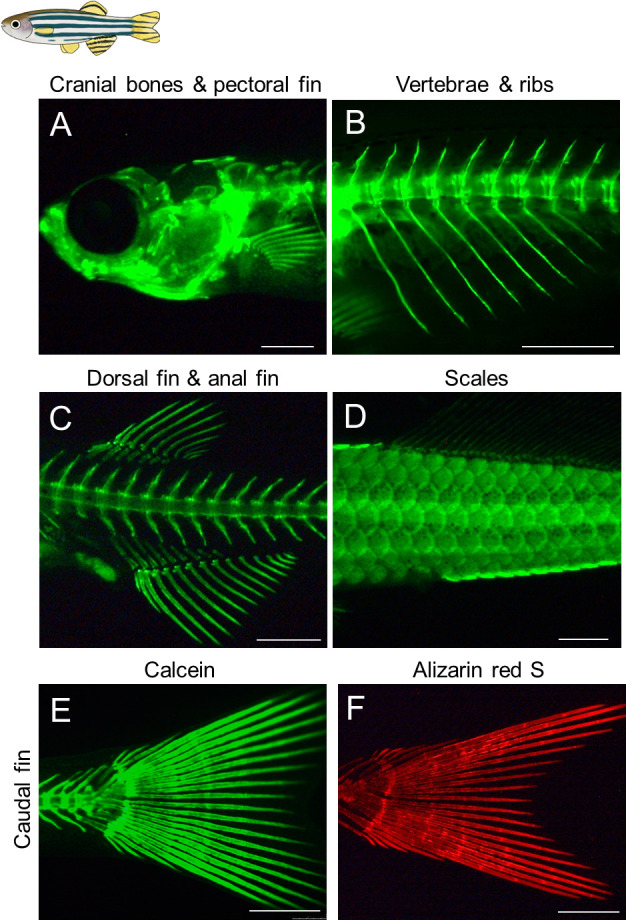
Visualization of the calcified bones in live juvenile zebrafish. (A–E) Calcein staining was performed for live zebrafish juveniles. (F) Alizarin red S staining. All the images are lateral views. Developmental stages of the stained fish are standard length (SL) 7.5 mm (A), SL 7.7 mm (B), SL 8.0 mm (C), SL 10.5 mm (D), SL 8.0 mm (E), and SL 7.8 mm (F). Scale bars: 500 μm.
    **Figure 3.**
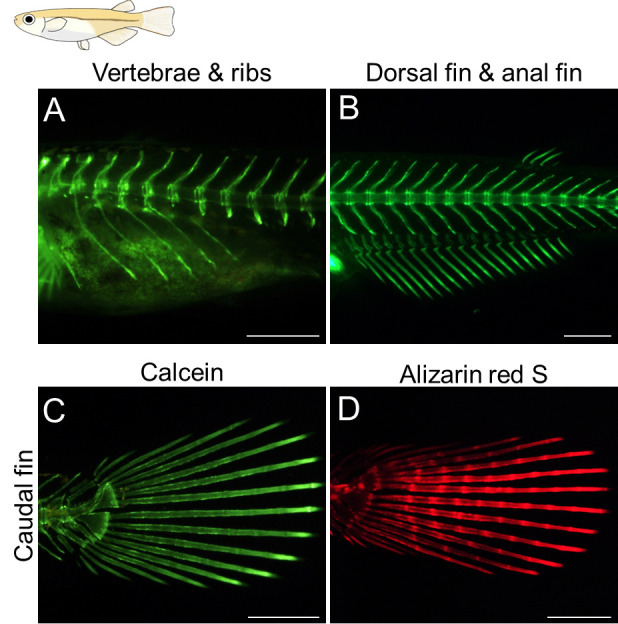
Visualization of the calcified bones in live medaka juveniles. (A–C) Calcein staining was performed for live medaka juveniles. (D) Alizarin red S staining. Developmental stages of the stained fish shown in (A–D) are stage 41 according to Iwamatsu’s paper [10]. All the images are lateral views. Scale bars: 500 μm.
  5. After finishing the photography, return the fish to the original 35 mm dish using a 3 mL transfer pipette without the tip. To supply oxygen to the fish and remove the viscous methylcellulose from its body, carefully apply water from the 35 mm dish onto the fish’s mouth and gills using a 3 cm transfer pipette. Once you have confirmed that the fish has recovered, cover the dish with a lid and proceed to anesthetize the remaining fish for analysis.
  6. If you wish to continue monitoring the progress, place each fish separately in a 35 mm dish or a 6-well dish and provide them with food and care. You can observe the calcified bone regularly by re-staining using calcein or alizarin red S, with several days between each observation.
  7. The procedures described above can be performed similarly for wild-type zebrafish (Figure 2), medaka (Figure 3), transgenic fish (Figure 4), and mutants (Figure 5).
    **Figure 4.**
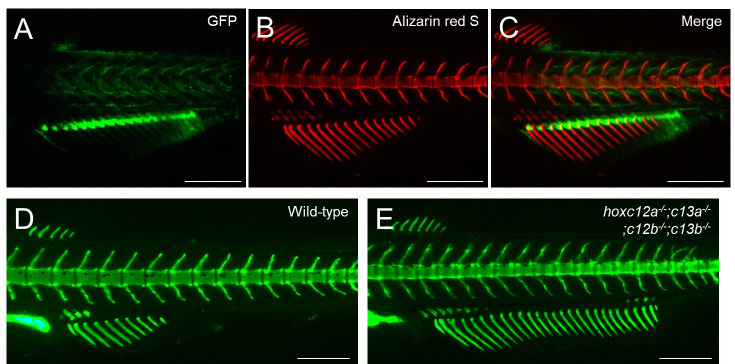
Example of alizarin red S staining in GFP-expressing transgenic zebrafish. (A–C) Transgenic juvenile zebrafish displaying a strong GFP signal in the proximal regions of the anal fin ray was stained with alizarin red S. The developmental stage of the stained fish is standard length (SL) 7.1 mm. Lateral views. Scale bars: 500 μm.
    **Figure 5.**
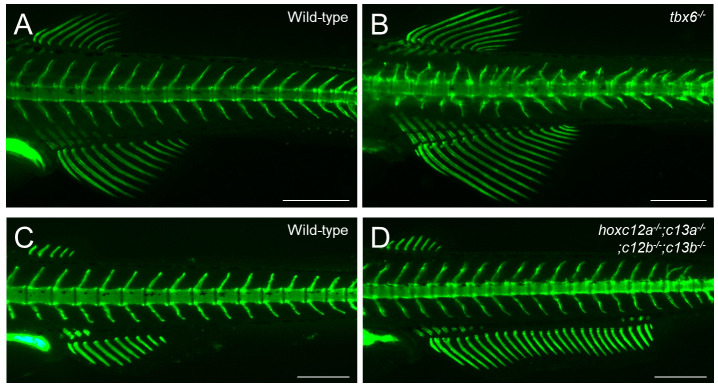
Examples of calcein staining in zebrafish mutant analysis. (A–D) Calcein staining was performed to distinguish the phenotypes of wild-type and mutant zebrafish. (A, B) Abnormal vertebral morphologies between wild-type and *tbx6* mutants. (C, D) The anal fin in *hoxc12a;c13a;c12b;c13b* mutants is expanded toward the posterior compared with that of the wild type. For details on the mutant phenotypes, see Ban et al. [13] and Adachi et al. [8]. The developmental stages of the stained fish are standard length (SL) 7.5 mm (A, B) and SL 6.9 mm (C, D). Lateral views. Scale bars: 500 μm.

## Data analysis

- **Measurements of the standard length of the stained fish by ImageJ**
  1. Use a photograph of a fish taken from the lateral side, showing the entire body, with a scale bar included.
  2. Open the image file in ImageJ software. FIJI, which is a version of ImageJ with additional plugins, can also be used.
  3. Click on the *Straight* button and measure the length of the scale bar as a reference.
  4. Click on the *Analyze* menu and select the *Set Scale* button.
  5. In the *Known distance*, enter the length of the measured scale bar and click on *OK*.
  6. Press the *Straight* button and measure the length of the stained fish from the tip of the snout to the posteriormost region of the body where caudal fin rays are inserted (Figure 6).
  7. Click on the *Analyze* menu and select *Measure*; then, the SL is obtained.
    **Figure 6.**
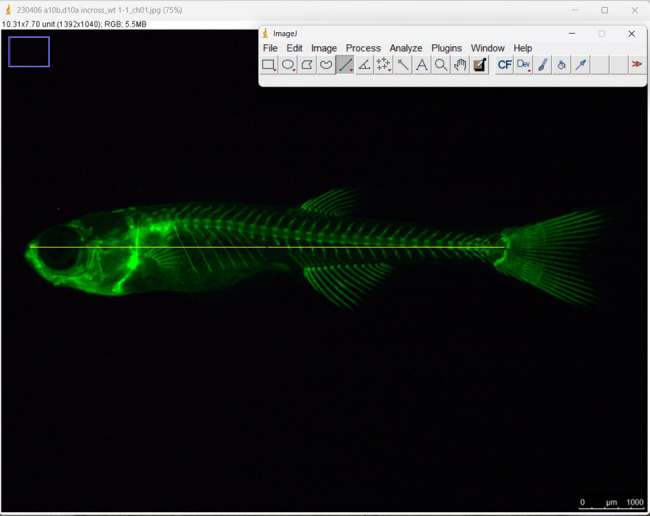
Measurement of the standard length (SL) in juvenile zebrafish by ImageJ. The yellow line corresponds to the standard length.
- **Measurements of the fluorescent signal intensities of the stained fish by ImageJ**
  1. Fluorescence intensities can be measured in specific areas of fish stained with calcein or alizarin red S. In this example, we will focus on a fin ray of the anal fin stained with calcein.
  2. Open the image you wish to analyze in ImageJ.
  3. Click on the *Image* menu and select *Color - Split Channels*. This will generate three images, each representing one of the RGB channels.
  4. For calcein-stained fish, use the image of the green channel. For fish stained with alizarin red S, use the image of the red channel.
  5. Select the *Segmented Line* tool and draw lines down the center of the area you wish to analyze (Figure 7A).
    **Figure 7.**
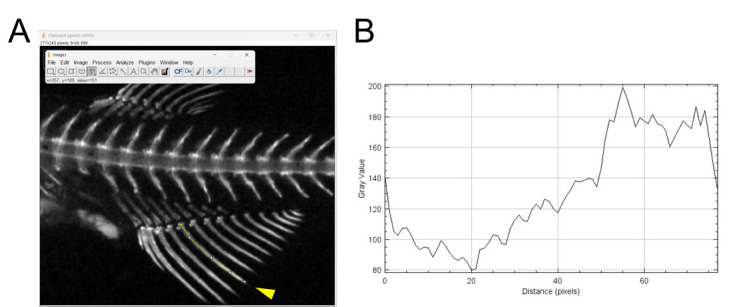
Measurement of signal intensities of fish stained with calcein by ImageJ. (A) Example of analyzing the fluorescence intensities of a fin ray using ImageJ. The yellow segmented lines indicate the fin ray to be analyzed (indicated by the arrowhead), extending from the proximal to the distal region. (B) A graph showing the signal intensities along the drawn lines.
  6. Click on the *Analyze* menu and select *Plot Profile*. A graph showing the signal intensities along the drawn lines will appear (Figure 7B).
  7. To ensure valid comparisons, use images taken under the same conditions.

## Validation of protocol

This protocol or parts of it has been used and validated in the following research article:

- Adachi et al. [8]. Teleost *Hox* code defines regional identities competent for the formation of dorsal and anal fins. *Proc Natl Acad Sci USA.* (Figure 2, panels C–E, J–L, Figure 3, panels A–C, G, H, N, O, Figure 4, panels H–K).]

## General notes and troubleshooting

1. This protocol outlines a method for visualizing calcified bone in live larvae and juveniles of zebrafish and medaka. For more examples obtained with this method, please refer to our previous papers [8,9,13,14].
2. When analyzing the whole skeletal structure in adult fish, we recommend using alizarin red and alcian blue staining or an X-ray micro-CT scan [15,16].
3. Chemical fixation with paraformaldehyde or other agents results in a loss of transparency in these larvae and juveniles, making it difficult to observe the fluorescent signals of their internal skeletons in fixed specimens. Additionally, when larvae and juveniles that have been stained are fixed, their transparency is similarly compromised, rendering them unsuitable for detailed observation of fluorescent signals.
4. Although pigment cells are observed during the developmental stages of staining, a sufficient fluorescent signal can be detected in their presence. Therefore, it is not necessary to prepare a treatment that inhibits the formation of pigment cells or to use specific strains in which pigment cells do not form.
5. In later developmental stages, sufficient fluorescent signal can be obtained by staining under the same staining conditions and at the same concentrations described above.
6. Zebrafish scale calcification begins around SL 8.0 mm, starting in the posterior portion of the body and gradually spreading throughout [17]. In medaka, the ossification of the scales is first observed at stage 42 [10]. Before scale calcification occurs, the internal skeletons, including the vertebrae, can be stained and visualized.
7. If the fish do not stain adequately, or if the staining process kills the fish, first check whether the reagents you have prepared are correctly made. If sufficient staining is still not achieved, we recommend increasing the staining time.
8. It is worth noting that stained bones can be observed at a higher resolution using confocal laser microscopy. However, compared to fluorescence stereomicroscopy, imaging with confocal microscopy requires more time and the fish must be kept stationary. Therefore, it is necessary to anesthetize the fish during imaging, and the survival rate of the fish after imaging is also lower than with the fluorescence stereomicroscope.
9. Although the present protocol is applied to zebrafish and medaka larvae and juveniles, the same method may be adaptable for other fish.

## References

[1] Furutani-Seiki M., Sasado T., Morinaga C., Suwa H., Niwa K., Yoda H., Deguchi T., Hirose Y., Yasuoka A., Henrich T., .(2004). A systematic genome-wide screen for mutations affecting organogenesis in Medaka, Oryzias latipes. Mech Dev. 121: 647-658. 15210174 10.1016/j.mod.2004.04.016

[2] Haffter P., Granato M., Brand M., Mullins M. C., Hammerschmidt M., Kane D. A., Odenthal J., F. J. M. van Eeden, Jiang Y. J., Heisenberg C. P., .(1996). The identification of genes with unique and essential functions in the development of the zebrafish, *Danio rerio* . Development. 123(1): 1-36. 9007226 10.1242/dev.123.1.1

[3] Fang J., Chen T., Pan Q. and Wang Q. (2018). Generation of albino medaka(*Oryzias latipes*) by CRISPR/Cas9. J Exp Zool B Mol Dev Evol. 330(4): 242-246. 29873175 10.1002/jez.b.22808

[4] Gagnon J. A., Valen E., Thyme S. B., Huang P., Ahkmetova L., Pauli A., Montague T. G., Zimmerman S., Richter C., Schier A. F., .(2014). Efficient Mutagenesis by Cas9 Protein-Mediated Oligonucleotide Insertion and Large-Scale Assessment of Single-Guide RNAs. PLoS One. 9(5): e98186. 24873830 10.1371/journal.pone.0098186 PMC4038517

[5] Irion U., Krauss J. and Nüsslein-Volhard C. (2014). Precise and efficient genome editing in zebrafish using the CRISPR/Cas9 system. Development. 141(24): 4827-4830. 25411213 10.1242/dev.115584 PMC4299274

[6] Nachtrab G., Kikuchi K., Tornini V. A. and Poss K. D. (2013). Transcriptional components of anteroposterior positional information during zebrafish fin regeneration. Development. 140(18): 3754-3764. 23924636 10.1242/dev.098798 PMC3754474

[7] Renn J. and Winkler C. (2009). *Osterix*‐mCherry transgenic medaka for in vivo imaging of bone formation. Dev Dyn. 238(1): 241-248. 19097055 10.1002/dvdy.21836

[8] Adachi U., Koita R., Seto A., Maeno A., Ishizu A., Oikawa S., Tani T., Ishizaka M., Yamada K., Satoh K., .(2024). Teleost *Hox* code defines regional identities competent for the formation of dorsal and anal fins. Proc Natl Acad Sci USA. 121(25): e2403809121. 38861596 10.1073/pnas.2403809121 PMC11194558

[9] Akama K., Ebata K., Maeno A., Taminato T., Otosaka S., K. Gengyo‐Ando, Nakai J., Yamasu K. and Kawamura A. (2020). Role of somite patterning in the formation of Weberian apparatus and pleural rib in zebrafish. J Anat. 236(4): 622-629. 31840255 10.1111/joa.13135 PMC7083572

[10] Iwamatsu T. (2004). Stages of normal development in the medaka Oryzias latipes. Mech Dev. 121: 605-618. 15210170 10.1016/j.mod.2004.03.012

[11] Kimmel C. B., Ballard W. W., Kimmel S. R., Ullmann B. and Schilling T. F. (1995). *Dev Dyn*. 203(3): 253–310. 10.1002/aja.1002030302 8589427

[12] Parichy D. M., Elizondo M. R., Mills M. G., Gordon T. N. and Engeszer R. E. (2009). Normal table of postembryonic zebrafish development: Staging by externally visible anatomy of the living fish. Dev Dyn. 238(12): 2975-3015. 19891001 10.1002/dvdy.22113 PMC3030279

[13] Ban H., Yokota D., Otosaka S., Kikuchi M., Kinoshita H., Fujino Y., Yabe T., Ovara H., Izuka A., Akama K., .(2019). Transcriptional autoregulation of zebrafish *tbx6* is required for somite segmentation. Development. 146(18): e177063. 10.1242/dev.177063 31444219

[14] Satoh K., Maeno A., Adachi U., Ishizaka M., Yamada K., Koita R., Nakazawa H., Oikawa S., Fujii R., Furudate H., .(2024). Physical constraints on the positions and dimensions of the zebrafish swim bladder by surrounding bones. bioRxiv. 10.1101/2024.07.04.602051 PMC11911126 39556020

[15] Walker M. and Kimmel C. (2007). A two-color acid-free cartilage and bone stain for zebrafish larvae. Biotech Histochem. 82(1): 23-28. 17510811 10.1080/10520290701333558

[16] Yamada K., Maeno A., Araki S., Kikuchi M., Suzuki M., Ishizaka M., Satoh K., Akama K., Kawabe Y., Suzuki K., .(2021). An atlas of seven zebrafish hox cluster mutants provides insights into sub/neofunctionalization of vertebrate Hox clusters. Development. 148(11): e198325. 10.1242/dev.198325 34096572

[17] Sire J., Allizard F., Babiar O., Bourguignon J. and Quilhac A. (1997). Scale development in zebrafish(*Danio rerio*). J Anat. 190(4): 545-561. 9183678 10.1046/j.1469-7580.1997.19040545.x PMC1467640

